# The preservation of right cingulum fibers in subjective cognitive decline of preclinical phase of Alzheimer’s disease

**DOI:** 10.3389/fnagi.2023.1223697

**Published:** 2023-10-30

**Authors:** Yu Sun, Yanan Qiao, Jing Guo, Wenjie Hou, Yaojing Chen, Dantao Peng

**Affiliations:** ^1^Department of Neurology, China-Japan Friendship Hospital, Beijing, China; ^2^State Key Laboratory of Cognitive Neuroscience and Learning, Beijing Normal University, Beijing, China

**Keywords:** Alzheimer’s disease, subjective cognitive decline, diffusion tensor imaging, cingulum, compensatory mechanism

## Abstract

**Introduction:**

Subjective cognitive decline (SCD) with a positive amyloid burden has been recognized as the earliest clinical symptom of the preclinical phase of Alzheimers disease (AD), providing invaluable opportunities to improve our understanding of the natural history of AD and determine strategies for early therapeutic interventions.

**Methods:**

The microstructure of white matter in patients showing SCD in the preclinical phase of AD (SCD of pre-AD) was evaluated using diffusion images, and voxel-wise fractional anisotropy (FA), mean diffusivity (MD), and axial and radial diffusivities were assessed and compared among participant groups. Significant clusters in the tracts were extracted to determine their associations with alterations in the cognitive domains.

**Results:**

We found that individuals with SCD of pre-AD may have subclinical episodic memory impairment associated with the global amyloid burden. Meanwhile, we found significantly reduced FA and λ1 in the right cingulum (cingulate and hippocampus) in AD dementia, while significantly increased FA and decreased MD as well as λ23 in the SCD of pre-AD group in comparison with the HC group.

**Discussion:**

In conclusion, increased white matter microstructural integrity in the right cingulum (cingulate and hippocampus) may indicate compensation for short-term episodic memory in individuals with SCD of pre-AD in comparison with individuals with AD and healthy elderly individuals.

## 1. Introduction

Alzheimer’s disease (AD) has been conceptualized as a cascade of neurodegenerative events leading to cognitive impairment. The accumulation of beta-amyloid (Aβ) plaques in the brain is considered to be the initial process preceding the onset of cognitive decline. Therefore, the AD continuum may include a long preclinical or prodromal period before clinical dementia. Subjective cognitive decline (SCD), which refers to a self-perceived cognitive decline in comparison with a prior normal aging status without objective impairment on standardized neuropsychological tests, has been recognized as the earliest clinical symptom of the AD continuum. Recent evidence has indicated that SCD is an increasingly important focus for the early diagnosis of AD. SCD is considered clinically heterogeneous and may depend on the individual’s psychological condition. Thus, identifying individuals with SCD in the preclinical phase of AD (SCD of pre-AD) by observing pathological changes using neuroimaging techniques provides a valuable opportunity to improve the existing understanding of the natural history of AD and determine strategies for early therapeutic interventions.

Microstructural lesions of associative fiber tracts in AD are likely rooted in primary neuron loss in gray matter regions, but also reflect primary white matter damage such as myelin breakdown [for review, see (Caso et al., 2016)]. Diffusion tensor imaging (DTI) is a non-invasive magnetic resonance imaging (MRI) quantitative technique used to study the microstructure of white matter (WM), and has been widely applied in research on the AD continuum. DTI metrics, including fractional anisotropy (FA) and axial (λ1), radial (λ23), and mean diffusivity (MD), can sensitively reflect changes in the WM microstructure, such as axonal injury, demyelination, necrosis, or edema (Shaikh et al., 2018). Many studies have demonstrated the loss of WM integrity in AD, starting from the preclinical phase (van Leijsen et al., 2018; Brueggen et al., 2019; Fogel et al., 2021; Chao et al., 2022). However, a previous study that included 179 individuals with pre-AD showed a nonlinear relationship between the WM microstructure and the regional amyloid burden. In particular, the study observed that increased FA and decreased MD were related to a lower amyloid burden, and subsequently, decreased FA and increased MD were related to a higher amyloid burden. The most conspicuous association was found between the Aβ burden in the precuneus and the FA and MD in the body of the corpus callosum (CC), which reflected multiple stages of axonal damage in the early amyloid deposition stage (Collij et al., 2021). Thus, the potential alterations in WM microstructure in Aβ-positive SCD (SCD of preclinical AD) have not been widely acknowledged and need to be studied further.

Amyloid brain positron emission tomography (PET) is a powerful tool for investigating AD pathology. Currently, by computing the standard uptake value ratios (SUVRs), the positive presence (i.e., values above the threshold of the global SUVRs) of ^18^F-florbetapir, which binds to Aβ plaques in the brain, can be evaluated to facilitate an antecedent diagnosis of the preclinical phase of AD (Collij et al., 2022). An SUVR threshold ≥1.17 was usually used to indicate pathological Aβ deposition, and has been shown to be associated with AD on the basis of ante-mortem PET data and post-mortem neuropathology data. Similarly, an SUVR threshold >1.08 was used to forecast the presence of any identifiable Aβ deposition because this was the upper limit from a set of cognitively normal middle-aged adults who did not carry the risk genetic apolipoprotein E (Fleisher et al., 2011). More generous criteria may recognize individuals in the ultra-early stage of Aβ accumulation, providing a time window that might be especially responsive to preclinical amyloid-modifying treatments for AD (Thal et al., 2015; Villeneuve et al., 2015).

In the present study, we intended to investigate the possible microstructural alterations in the WM in SCD of pre-AD patients who had a lower amyloid burden than the conventionally defined “amyloid positive” status, which may actually correspond to a relatively late stage of amyloid pathology. We aimed to provide new insights into previous studies that may have failed to explore the potential compensation in the ultra-early stages of SCD in preclinical AD.

## 2. Methods

### 2.1. Participants

We recruited 267 patients with cognitive impairment from the memory clinic of the China-Japan Friendship Hospital and 43 healthy controls (HCs) from the local community between October 2017 and October 2018. The inclusion and exclusion criteria were the same as those used in our previous study (Qiao et al., 2022). All participants underwent a standardized clinical evaluation, including assessments of medical history, physical examination, blood tests, a battery of neuropsychological assessments, and multi-modality MRI scans.

A total of 123 right-handed Han Chinese elderly individuals who completed ^18^F-florbetapir Aβ-PET were included in the present study. The diagnosis of SCD of pre-AD was based on the following research criteria for SCD proposed by SCD-I (Jessen et al., 2014): (1) self-experienced persistent decline in memory unrelated to an acute event; (2) neuropsychological performance on tests used to classify MCI were within the standardized norms and not meeting the criteria for MCI; (3) meeting the threshold for Aβ positivity. AD patients included those who met the National Institute of Neurologic Disorders and Stroke-Alzheimer Disease and Related Disorders Association (NINCDS-ADRDA) criteria (McKhann et al., 1984), met the threshold of Aβ positivity, and had a Clinical Dementia Rating (CDR) ≥ 0.5. The HCs were individuals with no complaints of cognition who showed normal neuropsychological performance with no Aβ deposition.

This study was approved by the Ethical Review Board of the China-Japan Friendship Hospital. All the participants provided written informed consent. The data for this study are available from the corresponding author upon request.

### 2.2. Neuropsychological assessments

All participants underwent a series of neuropsychological tests, including the Mini-Mental State Examination (MMSE) and Montreal Cognitive Assessment (MoCA), to assess general cognition. To assess the episodic memory domain, we evaluated the auditory-verbal learning test immediate recall (AVLT-I), delayed recall (AVLT-D), and total recall (AVLT-R), while the executive domain was assessed using the Trail Making Test Part B (TMT-B) and the Stroop Color and Word Test-right percent (SCWT-right). The category verbal fluency test (CVFT) was used to assess language ability, and the Symbol Digit Modalities Test (SDMT) was used to evaluate attention performance.

### 2.3. Amyloid PET

A total of 123 participants underwent Florbetapir F18 (AV45) PET using a Discovery TM PET/CT Elite scanner (General Electric) at the Beijing Tiantan Hospital, Capital Medical University. Florbetapir uptake in each cortical region was normalized to the uptake in the whole cerebellum to calculate the SUVR; this approach has been shown to yield good correlation with changes in amyloid pathology (Joshi et al., 2015). The cerebellum has been identified to be relatively spared from the Aβ plaque deposition in AD, validating the use of a cerebellum-based threshold for Aβ positivity (Joshi et al., 2012; Landau et al., 2013) and leading to the whole cerebellum being widely used as a reference region for florbetapir PET SUVR calculation (Johnson et al., 2013). The definition of Aβ positivity (Aβ+) was based on semi-quantitative assessments (SUVR >1.08) (Fleisher et al., 2011). SUVR was calculated using the cerebellar gray matter reference region to normalize the mean activity from 50 to 70 min.

### 2.4. Standard MRI acquisition

All MRI examinations were performed using a 3.0 T Siemens Tim MRI scanner at the Imaging Center for Brain Research, Beijing Normal University. The interval between amyloid PET and MRI did not exceed two weeks. We acquired T1-weighted, T2-weighted, fluid-attenuated inversion recovery (FLAIR), and DTI images from all participants. Anatomical MRI scans and images were visually inspected for apparent artifacts by two radiologists who provided almost the same reports. The detailed protocol for acquisition of all the MRI data are provided in the Supplementary material.

### 2.5. DTI imaging analysis

All diffusion MRI data were preprocessed and estimated by using a pipeline tool named “Pipeline for Analyzing braiN Diffusion imAges” (PANDA) (Cui et al., 2013). The raw data were preprocessed to correct for eddy current distortions and head motion. The b-matrix was reoriented for each participant to provide a more accurate estimate of the tensor orientations. Then, we mapped the diffusion data to the WM atlas JHU ICBM-DTI-81,^1^ which was overlaid on the WM skeleton of each participant in the template space such that each skeleton voxel could be categorized into one of the major tracts (Qiao et al., 2022). Voxel-wise statistical analysis of FA, MD, λ1, and λ23 at the skeleton voxels within each tract were performed by averaging the values within regions of the WM atlas with significant results. Here, we selected an FA threshold equal to 0.2 in order to restrict the analysis to more anisotropic regions with less significant inter-subject variability and partial-volume effects with gray matter.

### 2.6. Statistical analysis

The mean values of FA, MD, λ1 and λ23 at each skeleton tract according to the skeletonized map were extracted and used for statistical analysis in SPSS software package (version 24.0, IBM). To test group differences in demographic measures, we applied a one-way analysis of variance (ANOVA) for continuous data, and the chi-square test and Fisher’s exact test were used to compare categorical variables. Analysis of covariance (ANCOVA) using age, sex, and education as covariates was performed to evaluate group differences in neuropsychological assessments. Tukey’s correction for multiple comparisons was performed for *post-hoc* pairwise comparisons. Pearson’s correlation analyses were performed to explore the relationship between regional WM metrics and neuropsychological test results showing significant group differences (age, sex, and education were included as covariates). In all comparisons and correlations, the results were considered statistically significant at *p* < 0.05.

## 3. Results

### 3.1. Behavioral results

Among the 123 participants who underwent Aβ-PET examinations, patients showing cognitive impairment according to the neuropsychological assessments as well as Aβ positivity (Aβ+) were categorized into the AD group (*n* = 28), and those showing Aβ positivity with cognitive performance within the normal range were categorized into the SCD of pre-AD group (*n* = 18). We excluded 46 β-amyloid negative (Aβ-) patients showing abnormal cognitive assessment results and included Aβ- individuals with intact cognitive performance as HCs (*n* = 31).

The demographic characteristics are summarized in Table 1. The three groups showed no significant differences in age, sex, and education level. However, they showed significant differences in APOE ε4 prevalence and SUVR values for Aβ-PET, with higher prevalence of APOE ε4 allele and increased Aβ deposition observed in the AD and SCD of pre-AD groups than in the HCs.

**Table 1:** Demographic results for all participants.

	AD (*n* = 28)	pre-AD (*n* = 18)	HC (*n* = 31)	*F*-value	*p*-value
Age (years)	74.00 ± 6.13	73.61 ± 5.88	71.90 ± 8.51	0.70	0.499
Education (years)	11.50 ± 4.48	13.25 ± 4.82	12.20 ± 4.06	0.81	0.449
Gender (M/F)	9/19	10/8	16/15	3.22	0.200
APOE ε4 (±, %)	17/10(63.0%)	10/5(66.7%)	5/24(17.2%)	15.40	<0.001*^a^^,^^b^
SUVr	1.28 ± 0.02	1.25 ± 0.03	0.87 ± 0.02	87.97	<0.001*^a^^,^^b^

Values are mean ± standard deviation or Nos. of participants. * is *p* < 0.05.

a is significantly difference between AD and HC group.

b is significantly difference between pre-AD and HC group.

The neuropsychological measurements of all the participants are shown in Table 2. The AD group showed significantly lower performance than the HCs in almost all neuropsychological tests. However, significant differences between SCD of pre-AD and HCs were only found in parameters of the episodic memory domain, such as total recall (AVLT-R), immediate recall (AVLT-I), and delayed recall (AVLT-D), suggesting that subtle episodic memory changes may occur in the preclinical phases of Alzheimer’s disease when other aspects of cognitive integrity are still widely preserved.

**Table 2:** Neuropsychological tests result for each group.

	AD (*n* = 28)	pre-AD (*n* = 18)	HC (*n* = 31)	*F*-value	Effect sizes (partial Eta squared)	*p*-value
*General cognition*						
MMSE	18.83 ± 0.99	25.21 ± 1.26	26.07 ± 0.93	15.11	0.31	<0.001^*,^^a^
MoCA	15.54 ± 0.95	21.00 ± 1.21	22.18 ± 0.90	13.29	0.28	<0.001^*^^a^
*Episodic memory*						
AVLT-I	8.88 ± 0.90	11.98 ± 1.13	15.45 ± 0.83	13.96	0.30	<0.001^*^^a^^,^ ^b^
AVLT-D	0.94 ± 0.46	2.14 ± 0.58	4.74 ± 0.43	18.42	0.36	<0.001^*^^a^^,^ ^b^
AVLT-R	0.96 ± 0.44	1.66 ± 0.55	3.85 ± 0.41	12.28	0.27	<0.001^*^^a^^,^ ^b^
R-O delay	8.41 ± 1.69	10.90 ± 2.02	15.21 ± 1.47	4.60	0.14	0.014^*,^^a^
*Spatial processing*						
R-O copy	26.47 ± 1.60	33.19 ± 2.07	32.27 ± 1.50	4.55	0.12	0.014^*^^a^
CDT	19.91 ± 1.29	23.81 ± 1.61	23.48 ± 1.24	2.53	0.07	0.088
*Executive function*						
Stroop C-right	35.06 ± 2.21	43.75 ± 2.66	46.59 ± 2.04	7.38	0.19	0.001^*^^a^^,^ ^c^
TMT-B	265.21 ± 20.30	238.70 ± 22.12	174.07 ± 17.06	6.30	0.18	0.003^*^^a^
*Language ability*						
BNT	19.14 ± 1.10	23.80 ± 1.38	23.29 ± 1.02	4.84	0.13	0.011^*^^a^
CVFT	24.72 ± 3.21	33.25 ± 4.02	39.74 ± 2.97	5.68	0.147	0.005^*^^a^
*Attention*						
SDMT	16.12 ± 2.18	22.05 ± 2.57	27.27 ± 1.85	7.42	0.20	0.001^*^^a^
TMT-A	121.12 ± 13.08	80.03 ± 15.14	70.24 ± 11.36	4.41	0.13	0.016^*^^a^

The group differences in behavior scores among the three groups were tested with ANCOVA adjusted for age, sex, and education. *p* < 0.05 was considered significant. * is *p* < 0.05.

a is significantly difference between AD and HC group.

b is significantly difference between pre-AD and HC group.

c is significantly difference between AD and Pre-AD group.

### 3.2. Comparison of WM skeleton voxel-wise metrics

**Figure 1:**
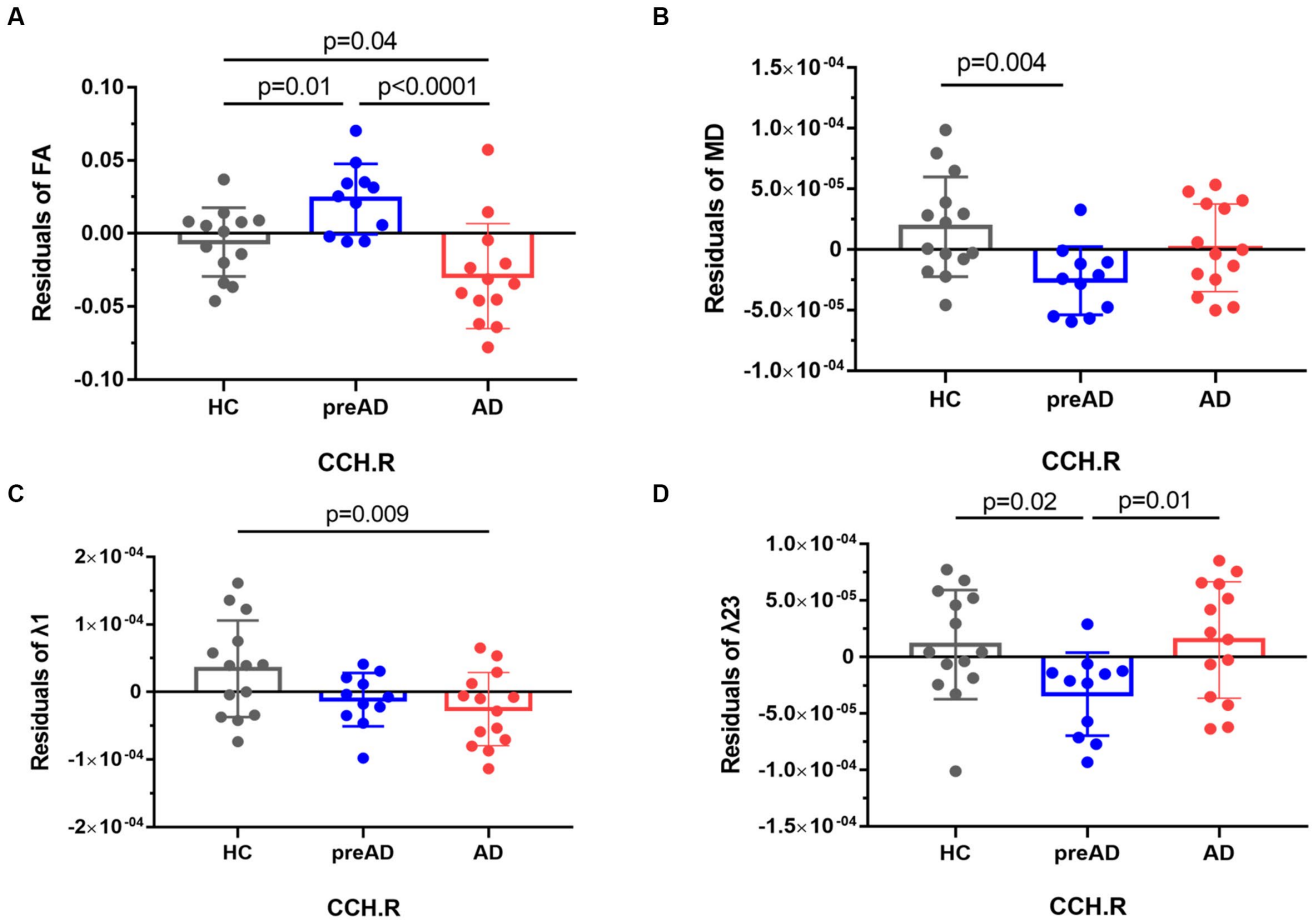
The comparison of voxel-wise white matter metrics in the right cingulum among all groups.

Among the AD, SCD of pre-AD, and HC groups, we found significant differences in FA and λ1, as well as MD and λ23 in the right cingulum (cingulate and hippocampus, CCH) (FWE-corrected, *p* < 0.05, see Figure 1). In the *post-hoc* analysis comparing the AD and HC groups, patients with AD dementia showed significantly reduced FA (*p* = 0.04, see Figure 1A) and λ1 (*p* = 0.009, see Figure 1C) in the right CCH, indicating disruption of white matter (WM) integrity in AD. Intriguingly, we found significantly increased FA (*p* = 0.01, Figure 1A) and decreased MD (*p* = 0.004, Figure 1B) and λ23 (*p* = 0.02, Figure 1D) in the SCD of pre-AD group in comparison with the HC group.

### 3.3. Correlations between DTI metrics and neuropsychological performances

In comparisons of the relationships between the significantly different WM metrics and memory scores among all participants (Table 3), a positive correlation was observed between the AVLT-immediate recall scores and both the FA (*r* = 0.473, *p* = 0.003) and λ1 (*r* = 0.379, *p* = 0.019) values of the right CCH. Moreover, the decrease in AVLT-immediate recall scores was associated with higher values of λ23 in the right CCH (*r* = −0.42, *p* = 0.009).

**Table 3:** Correlation of AVLT and global amyloid or white matter tracts.

	AVLT-I		AVLT-D		AVLT-R	
	*R*	*p*-value	*R*	*p*-value	*R*	*p*-value
*CCH.R*						
FA	0.473*	0.003	0.309	0.059	0.323	0.048
MD	−0.148	0.376	−0.031	0.855	0.001	0.998
λ1 (10^−3^)	0.379*	0.019	0.234	0.157	0.311	0.058
λ23 (10^−3^)	−0.420*	0.009	−0.187	0.260	−0.199	0.230
*Aβ*						
Suvr	−0.468*	0.005	−0.540*	0.001	−0.530*	0.001^c^

The group differences in measures of FA, MD, λ1 and λ23 in white matter tracts among the three groups were tested with ANCOVA adjusted for age, sex, education, WMH and lacunas. *p* was < 0.05 after Bonferroni correction.

a is significantly difference between pre-AD and H group.

b is significantly difference between ADCI and H group.

c is significantly difference between ADCI and Pre-AD group. CCH, cingulum (cingulate gyrus and hippocampus); ILF, inferior longitudinal fasciculus; L, left; R, right.

When we further explored the association between Aβ deposition and cognitive performance, we found a significant negative correlation between SUVR values and episodic memory performance. Higher Aβ deposition was associated with decreased AVLT-immediate recall (−0.468, *p* = 0.005), delayed recall (*r* = −0.54, *p* = 0.001) and total recall (*r* = −0.53, p = 0.001).

## 4. Discussion

Our current study demonstrated three main findings: (1) in the SCD of pre-AD stage, patients may have subclinical episodic memory impairment; (2) increased WM microstructure integrity in the right cingulum (cingulate and hippocampus) may suggest compensation for short-term episodic memory in patients with SCD of pre-AD in comparison with patients with AD and healthy elderly individuals; and (3) preservation of right cingulum fibers could not resist the episodic memory decline, which may be attributable to the global amyloid burden.

Most studies have reported that the preclinical phase of AD shows subtle deficits in verbal episodic learning and memory tasks (Collie and Maruff, 2000), while other cognitive abilities (e.g., executive function and language) seem to remain intact. Our previous study observed higher spontaneous brain functional activity in patients with SCD than in HCs, which was associated with better verbal episodic memory scores, indicating a potential compensatory mechanism in the ultra-early phase of AD (Sun et al., 2016). In the present study, we used voxel-wise analysis to explore the microstructural alterations of the WM in the preclinical stage of AD and found that in comparison with the HC and AD groups, patients with SCD of pre-AD have increased FA and λ1 as well as decreased MD and λ23 in the right cingulum tracts across the cingulate gyrus and hippocampus. FA reflects the degree of directionality of cellular structures and MD is a directionally averaged measure of the apparent diffusion coefficient within WM fibers (Basser et al., 1994; Alexander et al., 2007). Both help to elucidate alterations in WM integrity. Furthermore, the diffusion tensor eigenvalues may be separated into components that describe the diffusivity parallel (λ1) or perpendicular (λ23) to the axonal tracts (Xue et al., 1999). Reduced FA and increased MD, which suggest disrupted WM integrity in the cingulum, have been observed in multiple studies on individuals in the high-risk or pre-dementia stages of AD (Bendlin et al., 2010; Smith et al., 2010; Luo et al., 2019). As a part of the Papez circuit (Papez, 1995), the cingulum is one of the main tracts associated with the entorhinal cortex, which plays an important role in memory function as it has direct connections with the medial temporal lobe. However, our results revealed subtle increases in white matter changes in the preclinical stage of AD that were not detectable using conventional MRI, suggesting an innovative compensation mechanism in the early course of AD. A recently published study that included data from two large, independent, ongoing longitudinal observational studies showed consistently increased global connectivity of the limbic regions across the two SCD cohorts (Jiang et al., 2023). The results showed significantly higher degree of connectivity values in the cingulate cortex, hippocampus, and amygdala in SCD in comparison with HC, which is frequently interpreted as a compensatory phenomenon (Catani et al., 2013), suggesting that vulnerable brain regions aroused excessively increasing connectivity.

A growing body of literature is now addressing the role of WM tract microstructural alterations in cognitively unimpaired individuals with elevated brain amyloid burden in the maintenance of memory representations. Previous studies in cognitively normal older populations have suggested a nonlinear association between alterations in WM microstructure and the amyloid burden. Wolf et al. found that increased FA as well as decreased MD were associated with higher deposition in the tracts of the CC, fornix, corona radiata, internal capsule, and cingulum at a relatively low whole-brain Aβ levels (Wolf et al., 2015; Dong et al., 2020). At a high amyloid burden, higher deposition was associated with decreased FA and increased MD. Similarly, Dong et al. observed increased FA in the genu of the CC, anterior corona radiata, and fornix when comparing individuals with an intermediate Aβ burden to those with a low Aβ burden (Dong et al., 2020). Furthermore, these differences decreased overall from individuals with an intermediate Aβ burden to those with a high Aβ burden. A more recent study showed similar results, indicating that increased FA and decreased MD were initially associated with a low amyloid burden, while decreased FA and increased MD were associated with a higher amyloid burden subsequently (Collij et al., 2021). Thus, the initial stage of Aβ deposition may involve compensatory processes that preserve cognitive functioning.

Evidence regarding the sustained preservation of cognition in the preclinical stage of AD during the initial phase of amyloid deposition is not unequivocal. Changes in cognition as well as functional connectivity, regional metabolism, and brain volume occur even in cases with subthreshold amyloid deposition (defined as a mean distribution volume ratio of amyloid lower than 1.07 or 1.3 independent of visual inspection) (Veitch et al., 2019). Even in healthy adults, early deposition of amyloid plaques in specific regions is related to a subclinical amyloid-related decline in episodic memory (Farrell et al., 2018), which is consistent with our findings showing that a significant negative correlation between SUVR values and episodic memory performance surpassed the possible WM compensatory mechanism. Although limited, these findings imply that the seemingly ambiguous relationship between amyloid pathology and cognitive impairment may be more obvious at the stage in which the amyloid starts driving the cascade, but before the appearance of clinically relevant cognitive impairment.

There are several limitations that should be addressed. Firstly, all presented data were cross-sectional. The SCD subjects were known as very early stage of AD continuum and may compromised of clinically heterogeneous conditions. A longitudinal study to develop AD is therefore warranted. Considering that we include amyloid PET which was an important tool for elucidating the pathophysiologic mechanisms in patients with SCD. Further longitudinal cohort with SCD of pre-AD is on-going and credited analyses should be conducted. In addition, though we counted on only WM microstructure indicators such as FA, MD, λ1, and λ23 in this study and did not have direct functional application to the same population of SCD, they provide a prospect that early detection using a brain biomarker may be possible. Future analyses incorporating complementary data from other relevant modalities (for example, electrophysiology and other imaging modalities such as functional MRI) may replenish the compensatory features of SCD we observed. In conclusion, the present study showed increased WM microstructure integrity in the right cingulum (cingulate and hippocampus), which may indicate compensation for short-term episodic memory in individuals with SCD of pre-AD compared to AD and healthy elders. These results provide an interesting target for further interventional studies. Moreover, we noticed that individuals with SCD of pre-AD may have subclinical episodic memory impairment, which is associated with the global amyloid burden, supporting the foundational theory of β-amyloid in AD.

## Data availability statement

The original contributions presented in the study are included in the article/Supplementary material, further inquiries can be directed to the corresponding author.

## Ethics statement

The studies involving humans were approved by the Ethical Review Board of the China-Japan Friendship Hospital. All the participants provided written informed consent. The studies were conducted in accordance with the local legislation and institutional requirements. The participants provided their written informed consent to participate in this study.

## Author contributions

The study concepts, design, and manuscript editing were conducted by YS, YQ, and DP. The literature research and manuscript preparation were conducted by YS and YQ. The clinical diagnosis were made by YQ and DP. The clinical assessments and data acquisition were conducted by JG and WH. The data analysis and statistical analysis were conducted by WH and YC. All authors contributed to the article and approved the submitted version.

## Footnotes

^1^http://cmrm.med.jhmi.edu/
