# Supplementary material for: The preservation of right cingulum fibers in subjective cognitive decline of preclinical phase of Alzheimer’s disease
Source: Front Aging Neurosci. 2023 Oct 30;15:1223697. doi: 10.3389/fnagi.2023.1223697 (PMC10642356; doi:10.3389/fnagi.2023.1223697)
Supplement: Supplementary file 1 [file Table_1.DOCX]

Supplementary Material

The Preservation of Right Cingulum Fibers in Subjective Cognitive Decline of Preclinical phase of Alzheimer’s disease (SCD of pre-AD)

Yu Sun^1^ †, Yanan Qiao ^1^ †, Jing Guo^1^, Wenjie Hou^2^, Yaojing Chen^2^ and Dantao Peng^1^*

^1^Department of Neurology, China-Japan Friendship Hospital, Beijing, China.

^2^State Key Laboratory of Cognitive Neuroscience and Learning, Beijing Normal University, Beijing, China.

*** Correspondence:**Dantao Peng, MD, Department of Neurology, China-Japan Friendship Hospital, Beijing, 100029, China.

Tel.: +86 1084205288; Fax: +86 1084205288; E-mail: [pengdantao2000@163.com](mailto:pengdantao2000@163.com).

\

**Data Acquisition**

All MRI data were acquired on a 3.0T Siemens Tim MRI scanner in the Imaging Center for Brain Research, Beijing Normal University. The time interval between MRI and amyloid PET was no longer than two weeks. T1-weighted, T2-weighted, fluid-attenuated inversion recovery (FLAIR) and DTI were obtained. Two different radiologists assessed the anatomical MRI scans and gave the nearly same reports.

Participants lay still with their heads fixed by straps and foam to minimize movement. The T1-weighted images were acquired using a magnetization prepared rapid gradient echo (MPRAGE) sequence with the following parameters: repetition time (TR) = 1,900 ms; echo time (TE) = 2.2 ms; flip angle = 9◦; acquisition matrix = 256 × 224; field of view (FOV) = 256 × 224 mm2; slice thickness = 1 mm; no gap 176 sagittal slices, and average = 1. The diffusion tensor imaging (DTI) data were acquired using a single-shot EPI sequence with the following parameters: TR = 11,000 ms; TE = 98 ms; flip angle = 90◦; acquisition matrix = 128 × 116; FOV = 256 × 232 mm2; slice thickness = 2 mm; no gap; 60 axial slices; and average = 3. Thirty non-linear diffusion weighting directions with b = 1,000 s/mm2 and one b0 image were obtained. All images were reviewed and the leukoencephalopathy and vascular comorbidity was evaluated by an experienced neuroradiologist.
