# Supplementary material for: The preservation of right cingulum fibers in subjective cognitive decline of preclinical phase of Alzheimer’s disease
Source: Front Aging Neurosci. 2023 Oct 30;15:1223697. doi: 10.3389/fnagi.2023.1223697 (PMC10642356; doi:10.3389/fnagi.2023.1223697)
Supplement: Supplementary file 2 [file Table_2.DOCX]

**The Preservation of Right Cingulum Fibers in Subjective Cognitive Decline of Preclinical phase of Alzheimer’s disease (SCD of pre-AD)**

***Supplementary Information***

**Yu Sun^1^ †, Yanan Qiao ^1^ †, Jing Guo^1^, Wenjie Hou^2^, Yaojing Chen^2^ and Dantao Peng^1^***

^1^Department of Neurology, China-Japan Friendship Hospital, Beijing, China.

^2^State Key Laboratory of Cognitive Neuroscience and Learning, Beijing Normal University, Beijing, China.

**†These two authors contributed equally to this work.**

*** Correspondence:**Dantao Peng, MD, Department of Neurology, China-Japan Friendship Hospital, Beijing, 100029, China.

Tel.: +86 1084205288; Fax: +86 1084205288; E-mail: [pengdantao2000@163.com](mailto:pengdantao2000@163.com).

**Supplemental Methods**

**Amyloid PET acquisition and process**

2.3.1 The Discovery TM PET/CT Elite scanner (General Electrics) which were used in a 3-dimensional scanning mode examining 47 slices with 3.25 mm thickness that spanned the entire brain. We used Florbetapir (AV45) as a marker of cerebral amyloid. Participants received intravenous of approximately 370 MBq (10 mCi) of Florbetapir F18 (AV45). A PET acquisition for Florbetapir F18 (AV45) was acquired in 10-15 minutes frames beginning 40-60 minutes post-injection. PET data were reconstructed using an ordered subset expectation maximization algorithm with weighted attenuation. Images were smoothed using a 5-mm Gaussian kernel with scatter correction, and evaluated prior to analysis of patient motion and adequacy of statistical counts.

2.3.2 We applied software SPM to analyze the data from PET-CT. The original CT data were filtered and extracted. Then, the T1 image was registered on the PET image and resampled to obtain the deformation field and its inverse transformation in the standard space. The PET image was registered to the standard space for spatial standardization. Correction for partial volume effects (PVEs) was done using the geometric transfer matrix (GTM) method (Rousset et al., 1998) as implemented in the PET-PVE12 toolkit in software SPM. Finally, the SUVR value of PET was standardized with cerebellar gray matter reference region, and the mean value of global brain region was extracted.

**DTI Data Acquisition**

All MRI data were acquired on a 3.0T Siemens Tim MRI scanner in the Imaging Center for Brain Research, Beijing Normal University. The time interval between MRI and amyloid PET was no longer than two weeks. T1-weighted, T2-weighted, fluid-attenuated inversion recovery (FLAIR) and DTI were obtained. Two different radiologists assessed the anatomical MRI scans and gave the nearly same reports.

Participants lay still with their heads fixed by straps and foam to minimize movement. The T1-weighted images were acquired using a magnetization prepared rapid gradient echo (MPRAGE) sequence with the following parameters: repetition time (TR) = 1,900 ms; echo time (TE) = 2.2 ms; flip angle = 9◦; acquisition matrix = 256 × 224; field of view (FOV) = 256 × 224 mm2; slice thickness = 1 mm; no gap 176 sagittal slices, and average = 1. The diffusion tensor imaging (DTI) data were acquired using a single-shot EPI sequence with the following parameters: TR = 11,000 ms; TE = 98 ms; flip angle = 90◦; acquisition matrix = 128 × 116; FOV = 256 × 232 mm2; slice thickness = 2 mm; no gap; 60 axial slices; and average = 3. Thirty non-linear diffusion weighting directions with b = 1,000 s/mm2 and one b0 image were obtained. All images were reviewed and the leukoencephalopathy and vascular comorbidity was evaluated by an experienced neuroradiologist.

**DTI Imaging analysis**

The labels of each WM tract were from the JHU ICBM-DTI-81 atlas (Mori et al. 2008). We found that significant differences in the right cingulum (cingulate and hippocampus, CCH) were in limbic tracts which were associated with typical AD deficits area. The labeled and skeletonized WM tract groups are described as follows: 1) commissural tracts, including splenium (SCC), body (BCC), and genu (GCC) corpus callosum; 2) brainstem tracts, including inferior (ICP), middle (MCP), and superior (SCP) cerebellar peduncles, pontine crossing tract (PCT), and medial lemniscus (ML); 3) projection tracts, including corticospinal tract (CST), cerebral peduncle (CP), internal capsule (subdivided into anterior (AIC), posterior (PIC), and retrolenticular (RIC) portions), and corona radiata (subdivided into anterior (ACR), superior (SCR), and posterior (PCR) portions); 4) limbic tracts, including cingulum (subdivided into cingulate (CGC) and hippocampal (CGH) portions), and fornix (FX); and 5) association tracts (corticocortical tracts connecting two cortical regions), including external capsule (EC), superior fronto-occipital fasciculus (SFOF), superior longitudinal fasciculus (SLF), sagittal stratum (SS), and uncinate fasciculus (UF). The FA, MD, λ1, and λ23 differences among major WM tracts or tract groups within the subjects were tested with ANOVA. The entire WM was used as a reference to examine the differential microstructural maturation of all tracts and tract groups.

**Supplemental References**

AKAIKE H editor. Information theory and an extension of the maximum likelihood principle, 2nd International Symposium on Information Theory.; 1973:Akademiai Kiado. 267-281 p.

Benjamini Y, Hochberg Y. 1995. Controlling the false discovery rate: a practical and powerful approach to multiple testing. Journal of the royal statistical society Series B (Methodological).289-300.

Burnham KP, Anderson DR. 2004. Multimodel inference: understanding AIC and BIC in model selection. Sociological methods & research. 33:261-304.

Clancy H, Dugdalei A, Rendle‐Shortt J. 1969. The diagnosis of infantile autism. Developmental Medicine & Child Neurology. 11:432-442.

Krug DA, Arick J, Almond P. 1980. Behavior checklist for identifying severely handicapped individuals with high levels of autistic behavior. Journal of Child Psychology and Psychiatry. 21:221-229.

Lord C, Rutter M, Le Couteur A. 1994. Autism Diagnostic Interview-Revised: a revised version of a diagnostic interview for caregivers of individuals with possible pervasive developmental disorders. Journal of autism and developmental disorders. 24:659-685.

Ouyang M, Cheng H, Mishra V, Gong G, Mosconi MW, Sweeney J, Peng Y, Huang H. 2016. Atypical age‐dependent effects of autism on white matter microstructure in children of 2–7 years. Human brain mapping. 37:819-832.
